# miR-875-5p exerts tumor-promoting function via down-regulation of *CAPZA1* in esophageal squamous cell carcinoma

**DOI:** 10.7717/peerj.10020

**Published:** 2021-01-05

**Authors:** Nan Kang, Yunwei Ou, Guangchao Wang, Jie Chen, Dan Li, Qimin Zhan

**Affiliations:** 1 State Key Laboratory of Molecular Oncology, National Cancer Center/National Clinical Research Center for Cancer/Cancer Hospital, Chinese Academy of Medical Sciences and Peking Union Medical College, Beijing, China; 2 Department of Pathology, Peking University People’s Hospital, Beijing, China; 3 Key laboratory of Carcinogenesis and Translational Research (Ministry of Education/Beijing), Laboratory of Molecular Oncology, Peking University Cancer Hospital & Institute, Beijing, China

**Keywords:** ESCC, miR-875-5p, SNP, *CAPZA1*, Down-regulate

## Abstract

Esophageal squamous cell carcinoma (ESCC) is one of the leading causes of cancer deaths worldwide. Currently, efficient genetic markers for diagnosis and treatment of ESCC are lacking. MicroRNAs (miRNAs) are global genetic regulators that control cancer gene expression by binding to the 3′untranslated regions (3′UTRs) of targeting mRNAs. In addition, miRNAs function as oncogenes or tumor suppressors in the progression of tumors. In the current study, we found that hsa-miR-875-5p (miR-875-5p) exhibited amplification in ESCC according to the TCGA database. Then, xCELLigence Real-Time Cell Analyzer (RTCA)-MP system and colony formation assays were employed to detect cell proliferationand colony formationability. The results showed that miR-875-5p promoted the proliferation ESCC cells. Subsequently, transwell results indicated that miR-875-5p promoted the invasion and migration of ESCC cells. Furthermore, we showed that miR-875-5p was able to bind to *CAPZA1*3’UTR, which contains the single nucleotide polymorphism (SNP), rs373245753, as reported in our previous study involving WGS and WES on ESCC. Subsequently, mRNA affinity pull-down assays verifiedthat the SNP disrupts miR-875-5p binding to *CAPZA1*. The current study is the first demonstration that miR-875-5p may function as an oncogene via down-regulation of *CAPZA1* expression in ESCC.

## Introduction

Esophageal cancer is one of the most aggressive cancers worldwide and is categorized into two subtypes: esophageal adenocarcinoma (EAC); and esophageal squamous cell carcinoma (ESCC) (Pennathur et al., 2013). Approximately 70% of the worldwide cases of ESCC occur in China, which is characterized by late detection and widespread metastases (Lin, Du & Wang, 2010; Pennathur et al., 2013). Like other common cancers, ESCC is a complex trait caused by genetic and environmental factors (Engel et al., 1991; Pennathur et al., 2013).

MicroRNAs (miRNAs) are a class of RNA molecules, approximately 22nt in length, that regulate gene expression through base pairing with the 3′UTRs of target mRNAs, resulting in mRNA cleavage or translation repression (Bartel, 2004; Lee & Vasudevan, 2013). Recent studies have shown that miRNAs are aberrantly expressed in many human cancers and function as oncogenes or tumor suppressors in the initiation, development, and metastasis of human carcinomas (Chen, 2005; Esquela-Kerscher & Slack, 2006; Nohata et al., 2013). It has been reported that *Let-7c* functions as a metastasis suppressor by targeting *MMP11* and *PBX3* in colorectal cancer (Han et al., 2012). Furthermore, miRNA-424 may function as a tumor suppressor in endometrial carcinoma cells by targeting *E2F7* (Quan et al., 2015). It has also been reported that miR-223-3p has a tumor-promoting role in head and neck squamous cell carcinoma (Bozec et al., 2017). Indeed, miR-875-5p is amplified 6% and 5% in EAC and ESCC from the TCGA database, respectively. There are several studies that have reported that miR-875-5p has an important role in tumor progression. Specifically, miR-875-5p promotes the invasion of lung cancer cells by inhibiting *SATB2* (Wang et al., 2017). Up-regulation of miR-875-5p induces poorly differentiated thyroid carcinoma (PDTC) cell proliferation and reduces apoptosis and radioiodine uptake in vitro by down-regulating *NIS* (Tang et al., 2019), however, a limited number of studies have focused on the function of miR-875-5p in ESCC. We conducted a series of functional assays to assess the effects of miR-875-5p on ESCC cell lines.

It has been reported that approximately 30% of human genes are regulated by miRNAs (Carthew, 2006). We used TargetScan (Lewis et al., 2003) and miRanda (John et al., 2015) databases to predict the downstream genes of miR-875-5p. Interestingly, we found that miR-875-5p targeted the *CAPZA1* 3′UTR containing the single nucleotide polymorphism (SNP), rs373245753, which we identified in our previous study on WGS and WES in ESCC (Song et al., 2014). *CAPZA1* encodes the α subunit of the F-actin capping protein, which binds to the barbed ends of actin filaments and modulates nucleation of actin polymerization (Tsugawa et al., 2018). Although *CAPZA1* has been reported to have ectopic expression in neuroblastoma, malignant melanoma, and gastric, liver, gastric, and breast cancers (Deng, Li & Zheng, 2017; Lee et al., 2013; Lo et al., 2007; Yu et al., 2011), little is known regarding the specific functions of *CAPZA1* in ESCC. The miRNAs that target the 3′UTR of the corresponding genes and the seed regions are highly-conserved evolutionarily (Chen & Rajewsky, 2006). SNPs in miRNA target sites in the 3′UTRs of mRNA represent a specific class of functional polymorphisms that may lead to gene expression alterations by disrupting miRNA-target gene binding (Zhang et al., 2011). Therefore, the purpose of this study was to determine whether miR-875-5p has a tumor-promoting function via down-regulation of *CAPZA1* and if the SNP has the potential disrupt the binding of miR-875-5p to *CAPZA1* in ESCC.

## Materials & Methods

### Cell culture and treatment

Human ESCC cell lines, YSE2, KYSE30, KYSE70, KYSE140, KYSE150, KYSE180, KYSE410, KYSE450, KYSE510, COLO680 were provided by Professor Y. Shimada of Kyoto University. YSE2, KYSE30, KYSE70, KYSE140, KYSE180, KYSE410, KYSE450, KYSE510, COLO680 were cultured in 90% RPMI1640 medium, with 10% fetal bovine serum and antibiotics, at 37 °C, 5% CO_2_; KYSE150 was cultured in the medium of 1:1 mixture of Ham’s F12 and RPMI-1640 containing 2% FBS and antibiotics at humidified atmosphere with 37°C, 5% CO_2_.

### Stable and transient transfection

Stable recombinant plasmids over-expressing *CAPZA1(T)* and *CAPZA1(G)* by lentiviral infection and matched control vectors were constructed by Obio Technology Co. Ltd (Shanghai, China). Plasmids over-expressing *CAPZA1 CDS* and matched control vectors pcDNA3.1were constructed by Generay Biotech Co. Ltd (Shanghai, China). The target genes [CDS-3′UTR(T) and CDS-3′UTR(G)] were inserted into the lentiviral vector (H145 pLenti-EF1a-EGFP-F2A-Puro-CMV-MCS). Luciferase pGL3 overexpression vectors [3′UTR(T) and 3′UTR(G)] and pGL3 control plasmids were constructed by Generay Biotech Co. Ltd (Shanghai, China). miR-875-5p mimics, miR-875-5p inhibitors, biotinylated miR-875-5p, and matched negative controls (NCs) were synthesized by Guangzhou RiboBio Co. Ltd. (Guangzhou, China). For stable transfection, cells at 70%–80% density were infected with lentivirus using Polybrene in serum-free medium, then stable expressing cells were selected with an optimal concentration of puromycin. For transient transfection or co-transfection, cells were transfected in serum-free medium using Lipofectamine2000 following the manufacturer’s protocol (Invitrogen, Carlsbad, California, USA) when 70%–80% confluence was reached. The cells were then harvested and followed by PCR or Western blot analysis.

### Cell proliferation assays

As previously descripted, cell proliferation was detected by using the xCELLigence Real-Time Cell Analyzer (RTCA)-MP system (Acea Biosciences/Roche Applied Science). 2,000 cells per well were suspended in E-Plate 96 (Roche Applied Science) with 100 µl medium and monitored in real time for 96 h. Cell index was read automatically every 15 min and the recorded growth curve were shown as cell index SD.

The colony formation assays were employed to test the colony formation ability of cells. 500–600 cells were seeded per well in 60 mm plates and grown for 10–14 days. Then cells were fix with methanol for 15 min and stained with 1% crystal violet-acetic acid solution for 20 min, colonies were visualized and quantitated by G:box (Syngene).

### Invasion and migration assays

A Matrigel™  invasion chamber (BD Biosciences, Bedford, MA, USA) was used to evaluate the migratory activity of ESCC cells. A total of 3 × 10^4^ cells/ml were suspended with serum-free RPMI basal medium and seeded in a 24-well chamber, then filled with 600–800 µl of RPMI-1640 medium with 20% FBS per well beneath the chamber. After overnight incubation, the cells which had migrated through the Matrigel™  membrane were fixed with methanol and stained with 1% crystal violet-acetic acid solution for 20 min.

For invasion assays, Matrigel™  matrix solution (1% Matrigel™  matrix diluted with serum-free RPMI basal medium) was prepared, the plates were incubated for 2 h, and then invasive activity was detected as described above. The number of invasive and migrating cells were quantified by fluorescence microscopy.

### DNA, RNA extraction and quantitative real-time PCR

Total DNA of cells was isolated by extraction kit according to the standard procedure (Tiangen, Beijing, China). Total RNA of cells was extracted with TRIzol reagent according to the standard procedure. Reverse transcription was performed by using Superscript II-reverse transcriptase kit (Invitrogen) following the manufacturer’s instructions. The real-time PCR was performed in triplicate with the Premix Ex Taq kit (Takara) and a 7300 real-time PCR system (Life Technologies) according to manufacturer’s instructions. Thermal cycling conditions were as follows: the first step, 95 °C for 30s and the ensuing 40 cycles, 95 °C for 5s, 65 °C for 31 s, and melt curve step: 95 °C for 15s, 65 °C for 1min, 95 °C for 15s. The PCR primers were provided by Invitrogen. *GAPDH* and *U6* mRNA was employed as an endogenous control for mRNA.

3′UTR primers for PCR:

F:5′ GCCTCATGGAATACTGTTGAACC 3′;

R:5′  GGATAGATCACTCTCTCACC 3′.

### Western blotting analysis

Transfected cells were collected using lysis buffer (1 ×PBS + 4% NP-40 + 0.2% proteinase inhibitor) and lysed by centrifugation at 12,000 rpm for 20 min at 4 °C. The supernatants were added with 2 × SDS–PAGE sample loading buffer and boiled for 5 min. Eighty micrograms of protein was fractionated by SDS–PAGE, then transferred to polyvinylidene difluoride membranes. The membranes were incubated with relative primary antibodies overnight at 4 °C, then secondary antibodies were incubated and quantified using Image Quant software (GE Healthcare Biosciences, Pittsburg, PA, USA).

### Biotin-labeled pull-down assays

Biotinylated miR-875-5p (Guangzhou RiboBio Co, Ltd, China) pull-down assay with target mRNAs was performed as described (Christoffersen et al., 2009; Shi, Han & Spivack, 2013; Wynendaele et al., 2010). Cells were transfected with control miRNA and biotin-labeled miR-875-5p and whole-cell lysates were harvested. Simultaneously, Streptavidin beads (Invitrogen) were coated with 10 µl per sample yeast tRNA (Ambion) and incubated 2 h at 4 °C. Then the beads were washed with 500 µl lysis buffer three times and resuspended with 50 µl lysis buffer. Sample lysates were mixed with pre-coated beads (50 µl per sample) and incubated with rotator overnight at 4 °C. Beads were pellet down by centrifugation at 5,000 rpm for 1min at 4 °C to remove unbound materials and then washed five times with 500 µl ice-cold lysis buffer. Next, 750 µl of TRIzol (Invitrogen) and 250 µl nuclease-free water was added to both input and pull-down samples to isolate RNA. Finally, qPCR was employed to detect the expression ratio.

### Statistical analysis

GraphPad Prism v.5 software (GraphPad Software, Inc., La Jolla, CA, USA) and R version 4.0.2 software were used for statistical analyses. Data of colony numbers and mRNA expression levels of *CAPZA1* were analysed by ANOVA. Data of colony formation, cell invasion and migration numbers of miR-875-5p, luciferase activity and the enrichment of *CAPZA1* mRNAs were analysed by Student’s *t*-test (two-tailed). Statistical differences are presented as the mean ± standard deviation (SD). A *p* value <0.05 was considered statistically significance.

## Results

### miR-875-5p promoted cell proliferation and metastasis of ESCC cells

**Figure 1:**
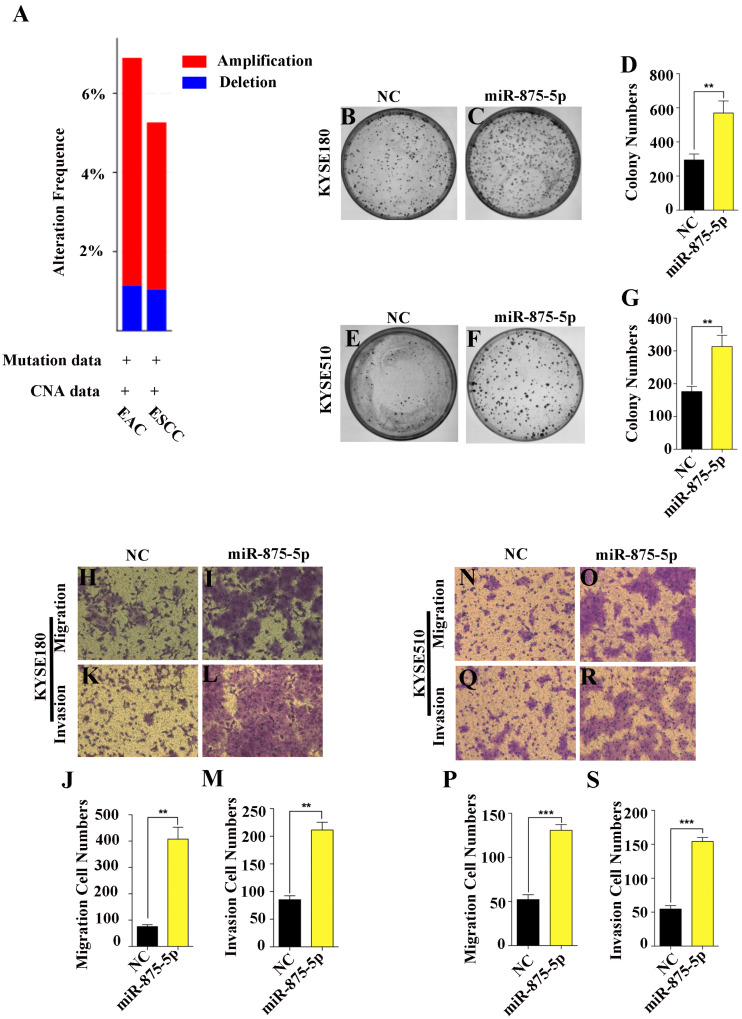
miR-875-5p promoted cell proliferation, migration and invasion of ESCC cells. (A) miR-875-5p encoding gene exhibits 6% and 5% amplification, while which was about 1% deletion in EAC and ESCC from TCGA and cBioProtal for Cancer Genomics database. (B, C) NC, miR-875-5p and miR-875-5p inhibitor were transfected into KYSE180 cells, and colony formation assays were employed to test the effect of miR-875-5p on ESCC cell proliferation. (D) Data analysis of colony numbers of NC, miR-875-5p and miR-875-5p inhibitor in KYSE180 cells, which were relative to miR-875-5p. (E, F) Colony numbers of KYSE510 cells. (G) Statistical analysis of colony numbers in KYSE510 cells. (H, I) Transwell assays were performed to detect the effect of NC, miR-875-5p and miR-875-5p inhibitor on KYSE180 cell migration. (J) Data analysis of migration in KYSE180 cells. (K, L) Transwell assays were employed to measure the influence of NC, miR-875-5p and miR-875-5p inhibitor on KYSE180 cell invasion. (M) Statistical analysis of invasive cell numbers of NC, miR-875-5p and miR-875-5p inhibitor in KYSE180 cells. (N, O) Migrating ability of KYSE510 cells. (P) Data analysis of migration in KYSE510 cells. (Q, R) Invasive ability of KYSE510 cells. (S) Statistical analysis of invasive cell numbers of KYSE510 cells. All experiments were performed at least three times and data were statistically analysed by ANOVA. ^∗^*p* < 0.05, ^∗∗^*p* < 0.01 and ^∗∗∗∗^*p* < 0.0001. Error bars indicate standard deviation (SD).

Given that miRNAs can function as oncogenes or tumor suppressors in the progression of human cancers (Chen, 2005; Esquela-Kerscher & Slack, 2006; Nohata et al., 2013), we first assessed the functions of miR-875-5p in ESCC cells. According to the TCGA and cBioProtal for Cancer Genomics database, we found that the gene which miR-875-5p encodes was amplified 6% and 5% in EAC and ESCC, respectively, while approximately 1% were deleted in EAC and ESCC (Fig. 1A). To determine the function of miR-875-5p in ESCC, we first introduced miR-875-5p mimics and miR-875-5p negative control(NC) into ESCC cells, then we performed colony formation and transwell assays in KYSE180 and KYSE510 cells. As shown in Figs. 1B to 1G, the expression of miR-875-5p significantly increased colony formation of ESCC cells compared with NC transfected cells, indicating that miR-875-5p promoted the proliferation ability of ESCC cells. In transwell assays, miR-875-5p markedly enhanced the migration and invasion of ESCC cells compared with NC group in KYSE180 and KYSE510 cells (Figs. 1H to 1S), which indicated that miR-875-5p facilitated metastasis of ESCC cells.

### miR-875-5p targeted the *CAPZA1* 3′UTR containing the T>G alteration at rs373245753

**Figure 2:**
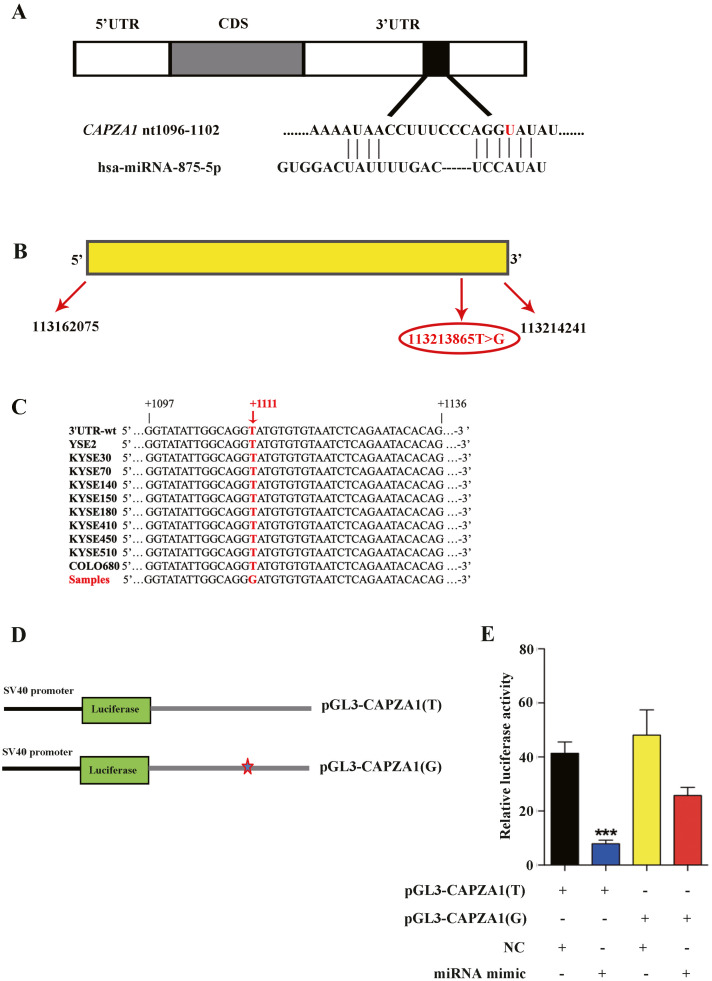
Identification of miR-875-5p base pairing with *CAPZA1 (T)*. (A) The sequence of miR-875-5p targeting to seed region of *CAPZA1* 3′UTR containing the reference allele (red). (B) The location of *CAPZA1* in genome and its single nucleotide variation in 3′UTR. (C) Sequencing of *CAPZA1* in ten ESCC cell lines. (D) The construct of *pGL3-CAPZA1(T)* and *pGL3-CAPZA1(G)* containing Renilla luciferase gene and full-length 3′UTR of *CAPZA1* gene. (E) *pGL3-CAPZA1(T)* and *pGL3-CAPZA1(G)* were co-transfected in parallel with NC and miR-875-5p mimic into KYSE180 cells and luciferase activity was performed to measure the targeting of miR-875-5p to *CAPZA1(T)* and *CAPZA1(G)*. Luciferase activity was measured and normalized to firefly luciferase. All experiments were performed at least three times and data were statistically analysed by two-tailed *t*-test. ^∗∗^*p* < 0.01. Error bars indicate SD.

Because SNPs may affect the interactions between miRNAs and their target genes, SNPs are thought to interfere with the mRNA expression of target genes by perturbing miRNA-mediated gene regulation (Chen et al., 2008). We used TargetScan database and predicted that miR-875-5p targeted the *CAPZA1* 3′UTR containing the T>G alteration at rs373245753 (Fig. 2A), which was show in our previous study involving WGS and WES on ESCC (Song et al., 2014). *CAPZA1* is located at chromosome 1 in humans with a length of 52167 bp (Fig. 2B). In addition, we employed a pair of PCR primers based on the 3′UTR and performed sequencing of the amplification products in 10 ESCC cell lines (YSE2, KYSE30, KYSE70, KYSE140, KYSE150, KYSE180, KYSE410, KYSE450, KYSE510, and COLO680). We showed that the T>G change at rs373245753 in the 3′UTR did not exist in these ESCC cell lines, indicating the need to heterogeneously-express the rs373245753 of 3′UTR in ESCC cells for further study (Fig. 2C). Then, the full length of *CAPZA1* 3′UTR fragments containing the rs373245753 T or G allele were cloned into the luciferase reporter vector, pGL3, to compare the luciferase activities between the two alleles (Fig. 2D). The pGL3 vectors containing the rs373245753 T or G allele of the *CAPZA1* 3′UTR were co-transfected in parallel with NC and miR-875-5p mimic to KYSE180 cells and luciferase activity was assayed. As shown in Fig. 2E, for the *pGL3-CAPZA1(T)* construct, the miR-875-5p mimic significantly reduced luciferase activity compared with NC, while no difference was observed in the *pGL3-CAPZA1(G)* group. Luciferase activity was measured and normalized to firefly luciferase and the reference is Ranilla luciferase. These findings indicated that miR-875-5p directly targeted the *CAPZA1* 3′UTR(T), and negatively regulated *CAPZA1* expression, while the variant allele attenuated these effects, thus allowing increased *CAPZA1* expression in the presence of this variant allele.

### *CAPZA1(T)* decreased the proliferation of ESCC cells and the function of *CAPZA1 CDS* was not reversed by the mimic of miR-875-5p

**Figure 3:**
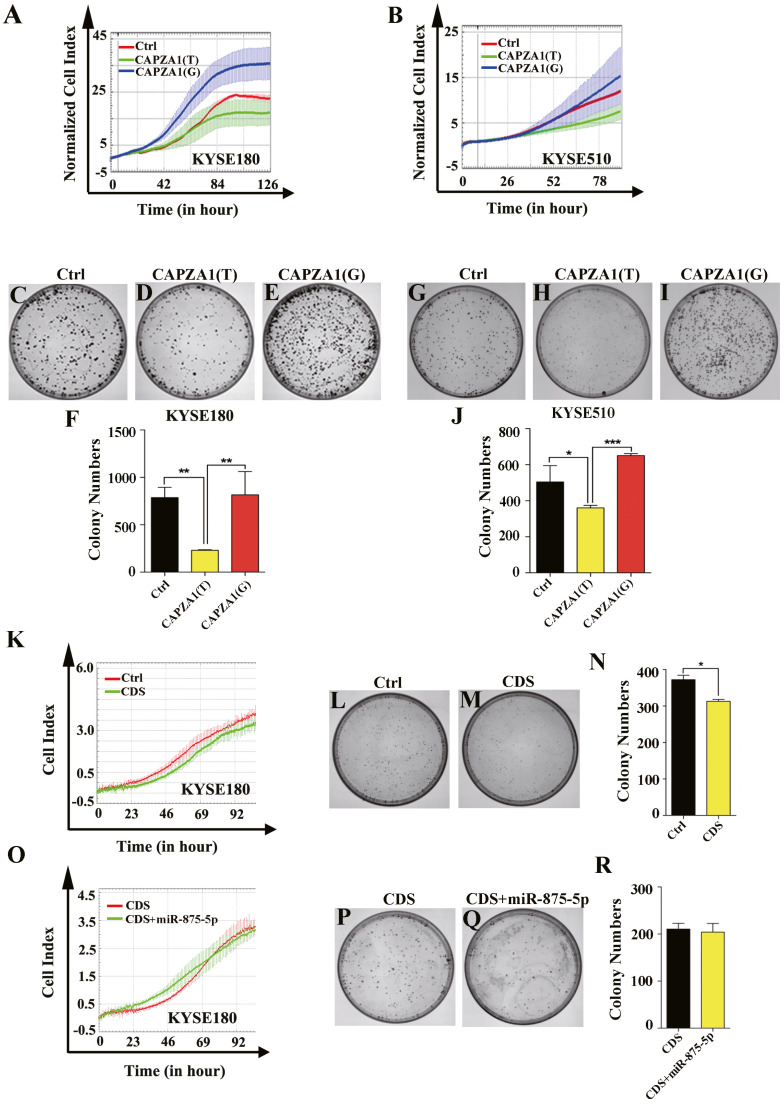
*CAPZA1(T)* decreased the proliferation of ESCC cells and the function of *CAPZA1 CDS* was not reversed by the mimic of miR-875-5p. (A) Growth curves were employed to measure the effect of *CAPZA1(T)* and *CAPZA1(G)* on cell proliferation in KYSE180 cells. (B) Growth curves were performed to measure the cell proliferation ability in stably overexpressed *CAPZA1(T)* and *CAPZA1(G)* KYSE510 cells. (C, D and E) Colony formation assays were employed to detected the effect of cell growth in stably overexpressed *CAPZA1(T)* and *CAPZA1(G)* KYSE180 cells. (F) Data analysis of colony numbers of control vector, *CAPZA1(T)* and *CAPZA1(G)* in KYSE180 cells, which were relative to *CAPZA1(T)*. (G, H and I) A total of 600 cells were seeded per well in 60 mm plates and colony formation assays were performed to test the influence of *CAPZA1(T)* and *CAPZA1(G)* on cell growth in KYSE510 cells. (J) Data analysis of colony numbers of KYSE510 cells. (K) Growth curves were selected to detect the function of *CAPZA1* CDS in KYSE180 cells. (L, M) 600 cells were seeded per well in 60 mm plates and colony formation were performed to detect the cell proliferation of control vector and *CAPZA1* CDS in KYSE180 cells. (N) Data analysis of colony numbers in KYSE180 cells. (O) Growth curves were employed to assess whether the cell proliferation of *CAPZA1* CDS was reversed by miR-875-5p in KYSE180 cells. (P, Q) 500 cells were seeded per well in 60 mm plates and colony formation were performed to measure cell growth ability of *CAPZA1* CDS and CDS+miR-875-5p in KYSE180 cells. (R) Statistical analysis of colony numbers in KYSE180 cells. All experiments were performed at least three times and data were statistically analysed by ANOVA and Student’s *t*-test (two-tailed). ^∗^*p* < 0.05, ^∗∗^*p* < 0.01 and ^∗∗∗∗^*p* < 0.0001. Error bars indicate SD.

Because the specific functions of *CAPZA1* in ESCC have not been reported, we first determined the functions of *CAPZA1(T)* and *CAPZA1(G)* in ESCC. Cells were stably overexpressed with *CAPZA1(T)* and *CAPZA1(G)* by lentiviral infection and we further assessed the functions in KYSE180 and KYSE510 cells. Cell proliferation ability was detected using an xCELLigence Real-Time Cell Analyzer (RTCA)-MP system (Roche, Basel, Switzerland) and growth curves showed that *CAPZA1(T)* reduced the rate of cell proliferation in KYSE180 and KYSE510 cell lines, whereas *CAPZA1(G)* substantially abolished these effects (Figs. 3A and 3B). Colony formation abilities in both KYSE180 and KYSE510 cells were markedly suppressed by *CAPZA1(T)* overexpression, but substantially increased in the *CAPZA1(G)* overexpression cell lines (Figs. 3C to 3J). Furthermore, we detected the function of *CAPZA1 CDS* in ESCC cells. Cells were overexpressed with *CAPZA1 CDS* and cell proliferation ability were assessed in KYSE180 cells. Cell growth curves and colony formation assays showed that *CAPZA1 CDS* significantly reduced the rate of cell proliferation in KYSE180 cells (Figs. 3K to 3N). Additionally, we wondered that whether the suppressive effect was reversed by miR-875-5p. Cells were co-transfected with *CAPZA1 CDS* and miR-875-5p, then cell growth curves and colony formation assays showed that the function of *CAPZA1 CDS* was not reversed by miR-875-5p (Figs. 3O to 3R). These results suggested that *CAPZA1 (T)* decreased the proliferation of ESCC cells and the function of *CAPZA1 CDS* was not reversed by the mimic of miR-875-5p.

### miR-875-5p differentially regulated *CAPZA1* expression in the presence of the SNP, rs373245753 T

**Figure 4:**
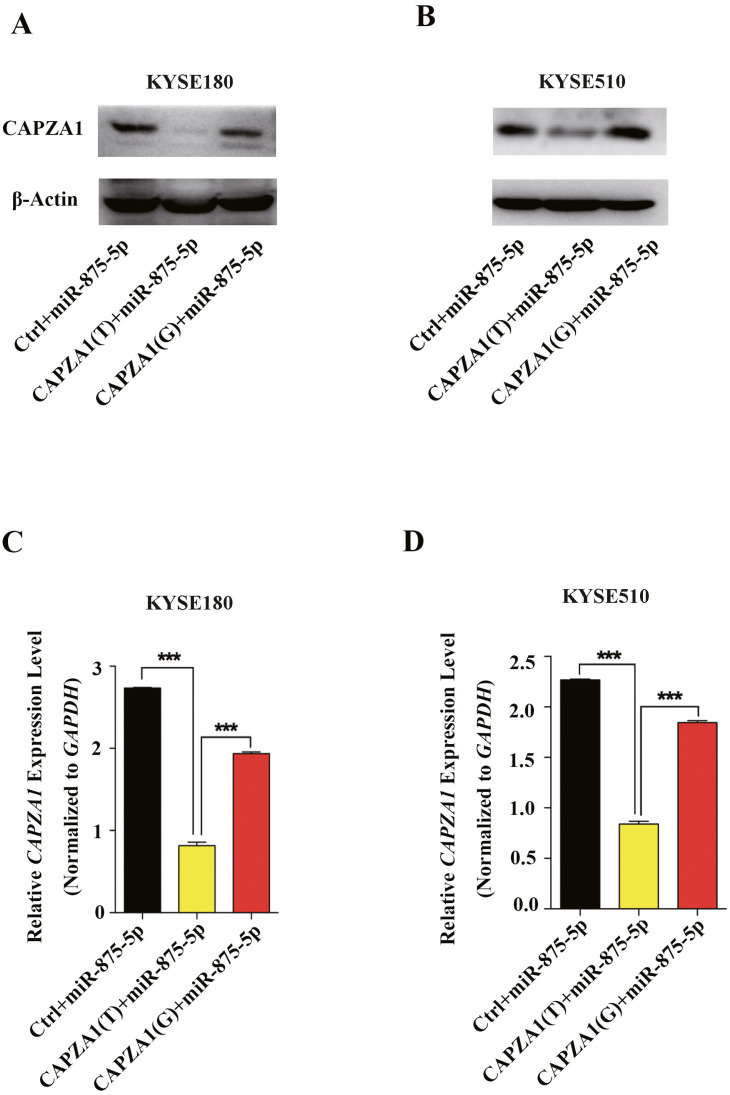
miR-875-5p targeted *CAPZA1(T)* and negatively regulated *CAPZA1* expression. (A) *CAPZA1(T)* and *CAPZA1(G)* plasmids were co-transfected with miR-875-5p mimic into KYSE180 cells and western blotting assays were performed to measure the regulation of miR-875-5p on the protein levels of *CAPZA1(T)* and *CAPZA1(G)* in KYSE180 cells. (B) Western blotting assays were performed to measure the regulation of miR-875-5p on *CAPZA1(T)* and *CAPZA1(G)* protein levels in KYSE510 cells. (C) qPCR assays were performed to assess the regulation of miR-875-5p on *CAPZA1(T)* and *CAPZA1(G)* mRNA levels in KYSE180 cells. (D) qPCR analysis of the regulation of miR-875-5p on *CAPZA1(T)* and *CAPZA1(G)* in KYSE510 cells. All experiments were performed at least three times and data were statistically analysed by ANOVA. ^∗∗∗^*p* < 0.001. Error bars indicate SD.

miRNAs can regulate gene expression and generate mRNA cleavage or translation repression by base pairing with their target mRNAs (Bartel, 2004; Lee & Vasudevan, 2013). To determine whether miR-875-5p preferentially regulates *CAPZA1* protein and mRNA levels of the *CAPZA1 (T)* and *CAPZA1(G)* transcripts, pGL3 vectors containing the rs373245753 T or G of the *CAPZA1* 3′UTR and miR-875-5p mimic were co-transfected into KYSE180 and KYSE510 cells. We found that the protein levels were markedly reduced in *CAPZA1(T)* cells compared to *CAPZA1(G)* and controls in KYSE180 and KYSE510 cells, which suggested that miR-875-5p might bind to *CAPZA(T)* and represses the protein expression of *CAPZA1* (Figs. 4A and 4B). In addition, qPCR assays showed that miR-875-5p could target *CAPZA1(T)* and suppressed *CAPZA1* mRNA levels in KYSE180 and KYSE510 cells (Fig. 4C and 4D). These observations indicated that miR-875-5p targets *CAPZA1(T)* and negatively regulates *CAPZA1* mRNA expression.

### The SNP, rs373245753 G, disturbed the binding of miR-875-5p and *CAPZA1* mRNA

**Figure 5:**
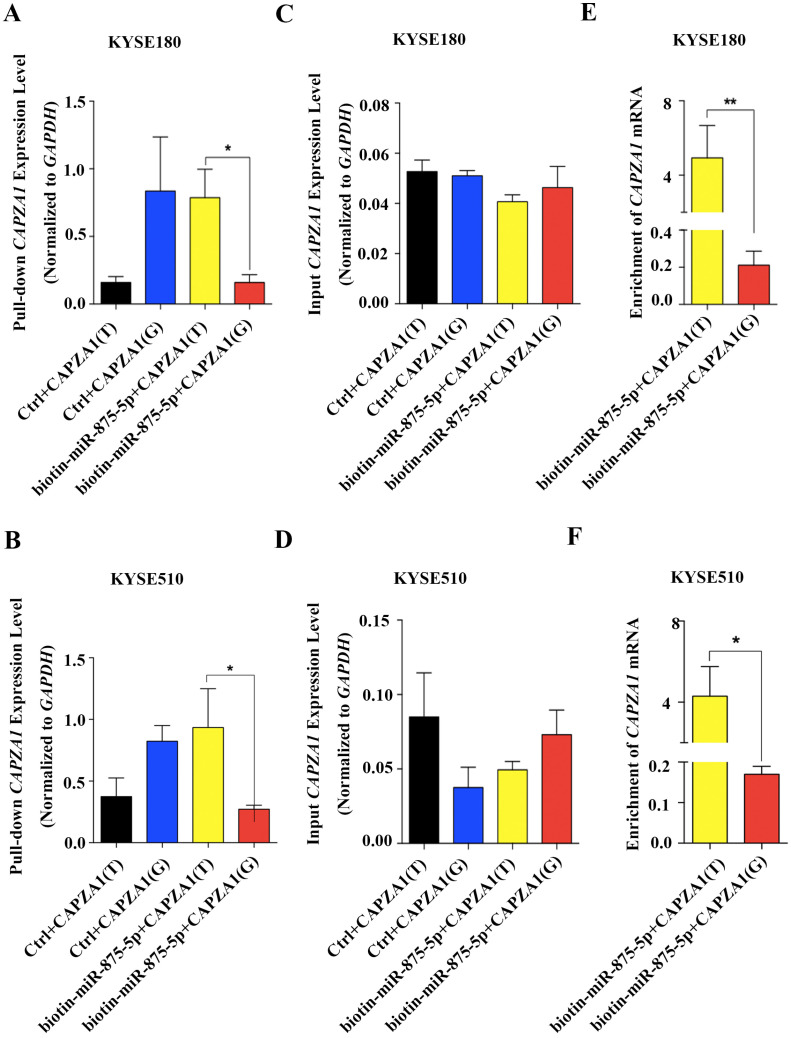
Association of miR-875-5p with *CAPZA1* mRNA. (A) Levels of total *CAPZA1* mRNA in the materials pulled down by biotin-miR-875-5p were measured by qPCR in KYSE180 cells (B). (C) Levels of input *CAPZA1* mRNA measured by qPCR in KYSE510 cells. (D) Levels of input *CAPZA1*mRNA in KYSE510 cells. (E) The enrichment of *CAPZA1(T)* and *CAPZA1(G)* mRNA pulled down by miR-875-5p were measured by qPCR in KYSE180 cells. (F) The enrichment of *CAPZA1* mRNA in KYSE510 cells. The enrichment of *CAPZA1* mRNAs binding to miR-875-5p was calculated as follows: mRNAs pull-down/control pull-down (X), mRNAs input/control input (Y), Fold binding = X/Y. Representative bar diagram from three independent experiments, each set of experiment was done in triplicates. Error bars mean SD and asterisk stands for statistically significant based on two-tailed Student’s *t*-test where *p* < 0.05.

Increasing evidence has revealed that SNPs in the 3′UTRs of genes targeted by miRNAs can disturb or obstruct miRNA binding and consequently influence regulation of target genes, which might be associated with cancer (Sethupathy & Collins, 2008). To determine whether the SNP, rs373245753 T>G, influences the binding of miR-875-5p and *CAPZA1* mRNA, RNA affinity pull-down assays were performed (Christoffersen et al., 2009; Shi, Han & Spivack, 2013; Wynendaele et al., 2010). The pGL3 vectors containing the rs373245753 T or G allele of the *CAPZA1* 3′UTR were co-transfected with control or biotin-labeled miR-875-5p into KYSE180 and KYSE510 cells. Cell lysates were incubated with streptavidin-coated beads. RNA was harvested from the pull-down materials and amplified with *CAPZA1* by qPCR. A biotin-labeled scrambled miRNA served as the control in these experiments. Firstly, qPCR assays were performed to measure the levels of pull-down and input *CAPZA1* mRNA in the materials pulled down by biotin-miR-875-5p in ESCC cell lines (Figs. 5A to 5D). We found that *CAPZA1(T)* was markedly elevated pulled down by biotin-miR-875-5p than *CAPZA1(G)* groups, which indicated that the SNP would disturbed the binding of miR-875-5p and *CAPZA1* mRNA (Figs. 5A and 5B). Subsequently, the levels of *CAPZA1* mRNA were significantly enriched in the pull-down material isolated from KYSE180 and KYSE510 cells following co-transfection biotin-labeled miR-875-5p with *CAPZA1(T)* compared to *CAPZA1(G)*. We obtained that *CAPZA1(T)* were more highly bound with biotin-labeled miR-875-5p than *CAPZA1(G)* (Figs. 5E and 5F). Taken together, these results illustrated that the SNP, rs373245753 T>G, disturbed the binding of miR-875-5p and *CAPZA1* mRNA.

## Discussion

ESCC has one of the highest incidences among cancers in China. Currently, there are the clinical approaches for early diagnosis and efficient therapies are limited due to the incomplete understanding of the underlying mechanism(s) of ESCC (Essadi, Lalya & Mansouri, 2015). Recent studies have demonstrated that miRNAs can influence almost every genetic pathway and regulate diverse biological processes, including the initiation and progression of ESCC (Chen, 2005; Esquela-Kerscher & Slack, 2006; Kim & Cho, 2010; Lu et al., 2005).

It has been reported that mutations or abnormal expression of miRNAs are associated with various cancers and miRNAs can function as tumor-promoting genes or tumor suppressors (Chen, 2005; Esquela-Kerscher & Slack, 2006; Nohata et al., 2013). According to the TCGA database, we found that miR-875-5p is amplified 6% with 1% deletion in ESCC. Although it has been confirmed that miR-875-5p exerts tumor suppressor function via down-regulation of EGFR in colorectal carcinoma (CRC) (Zhang et al., 2016), the specific functions and relevant machinery of miR-875-5p in ESCC have not been elucidated. This finding prompted us to conduct a series of functional assays to assess the effects of miR-875-5p in ESCC cells. We observed that miR-875-5p increased the ability of cells to proliferate, migrate, and invade. Then, we investigated the downstream gene of miR-875-5p using public databases, and unexpectedly found that miR-875-5p targeted *CAPZA1* 3′UTR containing the SNP, rs373245753 (Lewis et al., 2003; John et al., 2015), which was identified in our previous study involving WGS and WES in ESCC (Song et al., 2014).

There is mounting evidence that SNPs in miRNA-target gene interactions might affect miRNA-mediated gene regulation by disrupting the binding of miRNA to target mRNA (Zhang et al., 2011), and even contribute to cancer development (Landi et al., 2013; Harris, Ryan & Robles, 2010). For example, the SNP in the *KIT* 3′UTR disrupts a miR-221/222 binding site in gastrointestinal stromal tumors (Ravegnini et al., 2019). The SNP in miR-146a is associated with decreased expression of *ETS1* (Wei et al., 2014). The SNP in a miRNA-1827 binding site in *MYCL1* alters *MYCL1* expression, which is associated with small-cell lung cancer (Xiong et al., 2011). The miRNA binding site in the SNP affects *IL23R* expression and is associated with breast cancer (Wang et al., 2012). In this study, we verified that miR-875-5p could directly target the *CAPZA1* 3′UTR, leading to decreased *CAPZA1* expression, while the variant allele disturbed the binding of miR-875-5p to the *CAPZA1* 3′UTR. Furthermore, for the first time we showed that miR-875-5p functions as an oncogene by down-regulating *CAPZA1* in ESCC. Because the specific functions of *CAPZA1* in ESCC have not been elucidated, this study also demonstrated that *CAPZA1 (T)* negatively regulated the proliferation and metastasis of ESCC cells, while *CAPZA1(G)* abrogated the suppressive ability. Additionally, we demonstrated that *CAPZA1 CDS* alone can directly inhibit the malignant phenotype of esophageal cancer, while miR-875-5p couldn’t reverse the suppressive effect of CDS on malignant phenotype of esophageal cancer. These results suggested that the miR-875-5p directly targeted *CAPZA1* 3′UTR(T), and consistently, the SNP rs373245753 T>G could influence the binding of miR-875-5p and *CAPZA1* 3′UTR(T), which indicated that miR-875-5p has a tumor-promoting function by down-regulation of *CAPZA1* in ESCC*.*

In above, the current study first demonstrated that miR-875-5p promoted the proliferation and metastasis of ESCC cells, probably through down-regulation of *CAPZA1* expression. Moreover, the SNP, rs373245753, within the *CAPZA1* 3′UTR interfered with miR-875-5p binding to *CAPZA1* mRNA. Based on these findings, it is reasonable to propose that miR-875-5p has as a tumor-promoting gene in ESCC, which might highlight the potential of miRNA profiling in cancer diagnosis. Because ESCC is one of the most aggressive cancers with a very poor prognosis, it is important to clearly understand the specific genetic factors for the progression of ESCC, which may facilitate the development of diagnostic and therapeutic approaches.

## Conclusions

In conclusion, we demonstrated that miR-875-5p functions as a tumor-promoting gene via down-regulation of *CAPZA1* in ESCC cells. These findings highlight the importance of miR-875-5p in the development of ESCC and suggest the potential of this miRNA as a biomarker in cancer diagnosis.

## Additional Information and Declarations

### Data Availability

The following information was supplied regarding data availability: Data are available at Figshare: Kang, Nan (2020): PeerJ miRNA raw data.zip. figshare. Figure. https://figshare.com/articles/figure/Peer_J_miRNA_raw_data_zip/13083566.
